# Particle deposition, resuspension and phosphorus accumulation in small constructed wetlands

**DOI:** 10.1007/s13280-017-0992-9

**Published:** 2017-11-21

**Authors:** Pia Geranmayeh, Karin M. Johannesson, Barbro Ulén, Karin S. Tonderski

**Affiliations:** 1 Department of Soil and Environment, Swedish University of Agricultural Sciences, P.O. Box 7014, 75007 Uppsala, Sweden; 2 Swedish National Forensic Centre, 581 94 Linköping, Sweden; 3 Department of Physics, Chemistry and Biology, Linköping University, 581 83 Linköping, Sweden

**Keywords:** Constructed wetlands, Resuspension, Sediment accumulation, Sediment deposition

## Abstract

To improve understanding of phosphorus (P) retention processes in small constructed wetlands (CWs), we analysed variations in sediment deposition and accumulation in four CWs on clay soils in east-central Sweden. Sediment deposition (in traps) generally exceeded the total suspended solids (TSS) load suggesting that resuspension and wetland base erosion were important. This was confirmed by quantification of particle accumulation (on plates) (1–23 kg m^−2^ year^−1^), which amounted to only 13–23% of trap deposition. Spatial mean P concentrations in accumulated sediment on plates (0.09–0.15%) were generally similar to temporal mean P concentrations of particles in water (0.11–0.15%). Deposition/accumulation was minor in one wetland with high hydraulic load (400 m year^−1^), suggesting that such small wetlands are not efficient as particle sinks. Economic support for CWs are given, but design and landscape position are here demonstrated to be important for effective P retention.

**Electronic supplementary material:**

The online version of this article (10.1007/s13280-017-0992-9) contains supplementary material, which is available to authorized users.

## Introduction

Wetlands are often constructed to retain particles and associated phosphorus (P) transported from agricultural areas. Sedimentation is regarded as the main retention process for particles and associated P in such wetlands (Kadlec and Knight 1996) and the settling of sediment particles on the wetland bottom is defined as sediment deposition, while the vertical increase in the sediment surface is defined as accretion (Nolte et al. 2013). Sediment deposition rate increases with lower water velocity and longer water retention time (Johnston 1991) and could thus generally be expected to be higher in larger wetlands. In contrast, small wetlands with a low ratio wetland area to catchment area (A_w_:A_c_) may have a high hydraulic load (HL) and consequently low particle retention (Stephan et al. 2005). On the other hand, the particle load is also higher in smaller wetlands, which could lead to higher area-specific retention (Braskerud 2001a) and smaller wetlands are also cheaper to construct.

Like most retention processes, sedimentation is not permanent and particles that have been deposited are known to be resuspended by turbulence caused by fast-flow hydrological events, wind and wave action or bioturbation (Søndergaard et al. 2003). Braskerud (2003) studied particle retention in two wetlands and found that the area-specific retention of total suspended solids (TSS) increased with HL. Braskerud attributed this to increased transport from the catchment of more easily settled coarser soil particles at higher HL. A later study confirmed that the clay and silt particles both eroded and settled as aggregates (Sveistrup et al. 2008). However, in Sweden there are problematic soils with fine colloidal clay particles that can be transported a long distance before settling (Ulén 2004). Furthermore, the small particles are probably more prone to resuspension than silt-sized particles or larger aggregates. This means that a relatively low HL, lower than that in the study by Braskerud (2003), would probably be needed to retain clay particles with associated P. The optimum wetland size for high area-specific retention of both particles and P under Swedish climate and soil conditions is currently unclear.

Internal P loads from wetland sediments can reduce the function of wetlands as net sinks for particles and P (Pant and Reddy 2003; Palmer-Felgate et al. 2011). Internal erosion may be important, as demonstrated in shallow lakes (Bloesch 1994), and resuspension of previously settled material may have an impact on wetland function as a trap for soil particles and associated P (Barber and Quinn 2012). Resuspension is affected by water flow, e.g. variations in HL, and the density of the plant cover (Braskerud 2001b). In particular, variations in the inflow may be important, and have been quantified by, e.g. Siber et al. (2009) using a simple fast-flow index (FFI). In addition, the shape and depth of a wetland can be important factors for the magnitude of water velocity variations (Wörman and Kronnäs 2005).

This study examined seasonal and spatial variations in sediment deposition in traps and annual variations in sediment accumulation on plates in four small wetlands constructed on agricultural clay soils in order to reduce the particle and P load to nearby streams and lakes. Three were newly constructed. An earlier study demonstrated highly dynamic total suspended solids (TSS) and P retention in one of these wetlands (Kynkäänniemi et al. 2013). The objectives here were to (i) analyse the relationship between seasonal sediment deposition, estimated TSS load and HL; (ii) estimate sediment deposition and accumulation in different parts of the wetlands; (iii) evaluate the importance of resuspension and internal erosion for annual sediment accumulation and (iv) examine the relationships between P concentrations in TSS and in accumulated sediment.

## Materials and methods

### Site description and wetland design

**Fig. 1:**
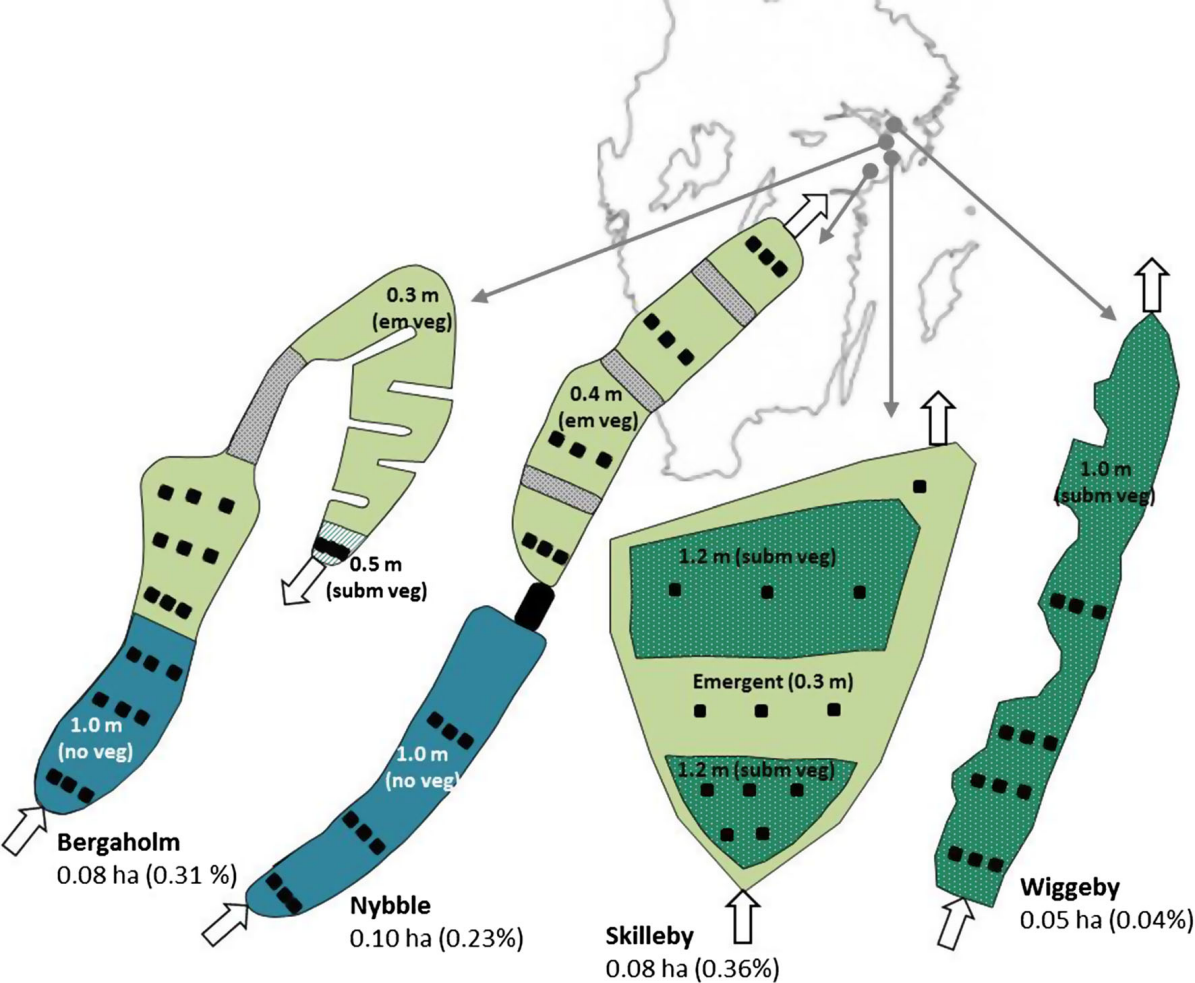
Shape with absolute area (ha) and size relative to catchment (%) of the Bergaholm (Ber), Nybble (Nyb), Skilleby (Ski) and Wiggeby (Wig) wetlands. Deep pond with no vegetation (blue), shallow emergent vegetation area (light green), deep pond with submerged vegetation (dark green dots) and shallow area with submerged vegetation (green stripes). The grey areas are overflow areas. Black squares indicate sediment sampling points (plates and traps) and arrows indicate inlet and outlets. In Ber, there were seven transects except in the first year, when the last transect closest to the Ber outlet was not yet installed

The four wetlands Nybble (Nyb), Bergaholm (Ber), Skilleby (Ski) and Wiggeby (Wig) were constructed in agricultural catchments within a 44 km radius within the same climate region in east-central Sweden (Fig. 1). The mean clay content in arable topsoil (0–20 cm) at the sites is 22–43%. All arable land in the region is tile-drained, but in Nyb and Wig the drain pipes discharge into an open ditch where the wetland is located (Table 1) (Johannesson et al. 2015). The four wetlands are of similar size (0.05–0.10 ha) but the A_w_:A_c_ ratio is only 0.04% for the Wig wetland (Table 1). Nyb, Ber and Wig are long and narrow, while Ski is wide and short and has a small length-to-width ratio (L:W) (Table 1). Their design also differs in depths and vegetation (Fig. 1). The Nyb and Ber wetlands were constructed according to Braskerud (2001a), with a deep (1 m) sedimentation basin followed by a shallow (0.3–0.4 m) area with emergent plants. The Wig wetland only consists of a deep basin, whereas in the Ski wetland, the deep basin is followed by a shallow area with emergent plants and a second deep section before the outlet. Information about vegetation in the wetlands is given in the Supplementary Material.

**Table 1:** Overview of the catchment and wetland size, mean clay and P content in agricultural soil, inlet type, water flow measurements, water sampling and analysis at inlets and outlets and phosphorus analysis of sediment accumulated on plates in Nyb, Ber Ski and Wig wetlands

Wetland	Nyb	Ber	Ski	Wig
Catchment				
Area (ha)	44	26	23	121
Clay content (0–20 cm) (%)	22	27	43	44
Soil P-AL (mg kg dry soil^−1^)	70	140	42	80
Wetland size				
Relative catchment (Aw:Ac) (%)	0.23	0.31	0.36	0.04
Length-to-width ratio (L:W)	11	14	2	7
Inlet type and water flow				
Inlet type	Open ditch	Drainage pipe	Drainage pipe	Open ditch
V notch	90°	90°	60°	–^a^
Water level load cell	Tedea-Huntleigh FX60		–	–^a^
Water level pressure transducer	–	–	PDCR 1830	–^a^
Water sampling	Composite flow proportional			Grab
Samples per year (mean)	22	22	18	22
Water analysis				
Suspended solids (SS)^c^	SS	SS	SS	SS
Turbidity (Turb)^d^	Turb	–	–	–
Total phosphorus (TP)^e^	TP	TP	TP	TP
Particulate P (PP)^f^	PP	PP	PP	PP
Dissolved reactive P (DRP)^g^	DRP	DRP	DRP	DRP
Sediment analysis				
Dry weight (DW)	DW	DW	DW	DW
Total phosphorus (TP_sed_)	TP_sed_	TP_sed_	TP_sed_	TP_sed_

^a^Daily runoff data (SMHI 2014) from the S-HYPE model (Strömqvist et al. 2012) for the larger river basin (ID 658404-1608848) in which Wig is located were adjusted to the Wig watershed area

^b^Only in the inlet. In periods of malfunction (January–September 2010), inflow for that period was estimated based on the Ber water inflow

^c^Analysed by weighting the filter-cake on filters with 0.2 μm pore diameter of (Schleicher & Schüll GmbH, Dassel, Germany)

^d^Recorded every 10 min with an online instrument (Scan:sensor nitro:lyser). Turbidity (FTU units) was calibrated from manual samples measured immediately after shaking the sample and by turbidimeter (Hach-Lange Company, Düsseldorf, Germany). Measurements took place in 2013/2015 as a quality control of the water sampling of SS

^e^Analysed within 4 days after storage at +4 °C using oxidation with K_2_S_2_O_8_ (ISO 15681-1 2003)

^f^Estimated as the difference between total P in filtered and unfiltered samples

^g^Analysed within 2 days after storage and after pre-filtration (EN ISO 6878 2004)

^h^The sediment collected from the plates was dried at 60 °C. The trap sediment was stored at approximately 6 °C before removal of visual detected litter and invertebrates. When the suspended material had settled, water was decanted off and the dry weight (60 °C) determined

^i^By digestion with 1 M HCl according to Svendsen et al. (1993), but using an autoclave (120 °C for 20 min)

### Sampling and analyses

Flow-proportional sampling of drainage water was carried out for three of the wetlands and grab sampling in the fourth (Table 1). Each flow-proportional subsample represented a certain amount of discharge, typically 0.15 L m^−2^. High peak flows were typically covered by at least four subsamples per hour and sampled water from such peaks always constituted the major part of composite samples. Composite water samples were collected in glass vessels (10 L) during two-week periods and stored in a refrigerator (4 °C). Water for chemical analysis was immediately sent to the Water Laboratory at the Department of Soil and Environment. Further information about water flow measurements and methods used for chemical analysis is given in Table 1.

**Fig. 2:**
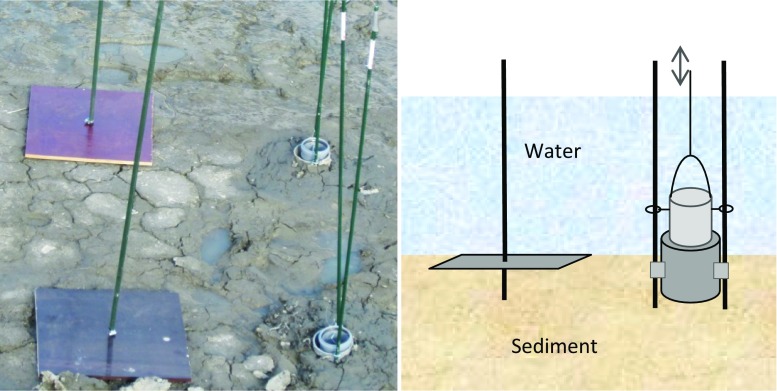
Sampling system: one sediment plate (40 cm × 40 cm in areas >1 m deep and 25 cm × 25 cm in areas <0.5 m deep) and trap (11 cm high and 7.5 cm diameter) was placed on the bottom of each wetland at each sampling point. Sediment accumulation was estimated using plates, which were exposed to resuspension, and sediment deposition using traps, which were not exposed to resuspension. Resuspended sediment was estimated as the difference between sediment traps and plates

Sediment plates (exposed to resuspension) made from plastic-covered plywood were anchored to the bottom (Fig. 2) in several transects (Fig. 1) and set level with the sediment surface. The plates were left for one year (summer to summer) and sampled from a defined area in the most undisturbed part of each plate (Johannesson et al. 2015). The accumulated amount of sediment (Accum) is here assumed to represent net annual particle retention. Along the same transects, plastic cylinder traps (not exposed to resuspension) were dug down into the sediment (Fig. 2), with the edge approximately 2 cm above the sediment surface adjacent to the plates. The intention was that the traps would act as collectors of suspended solids by lowering water turbulence inside the cylinders and this material is in the present study defined as deposition (Depos). The traps were sampled three times per year, representing different seasons: autumn (August-November), winter and snowmelt (November–April/May) and spring–summer (April/May–August). In the first pond in Nyb wetland, total sediment depth down to the firm sediment base created during wetland construction, was measured in year two using a measuring stick. For further sample handling and P analysis, see Table 1.

### Calculation and statistical analyses

Base Flow Index (BFI) was estimated in three yearly seasons, defined by the trap sampling periods, by dividing the flow that was exceeded 70% of the time by mean water flow. Fast-Flow Index (FFI) was estimated as [1 − BFI] (Siber et al. 2009). The wetland load of TSS (kg m^−2^ year^−1^) was calculated by multiplying the inflow (m^3^ d^−1^) by the flow-proportional sampled inflow concentration (mg L^−1^) for each sampling period and adding it up for each year. Flow-weighed TSS concentrations were calculated by dividing the annual load by the annual inflow. Phosphorus retention was estimated as the difference load_in_ − load_out_.

Sediment deposition (Depos) values from the three seasonal sampling occasions were added together for each trap to get the yearly amount. Deposition and accumulation (Accum) were then estimated to cover the entire wetland area by interpolating data at each sampling point using the Inverse Distance Weighted (IDW) method in ArcGIS 10.1 and the Zonal Statistics tool. If a sample was missing, the average dry weight for that transect and sampling occasion was used. Resuspension (Resusp) was calculated as the difference between the interpolated annual sediment deposition and accumulation, and relative resuspension (%) was expressed as a percentage of sediment deposition.

Possible correlations between sediment deposition, accumulation and resuspension and L:W, relative wetland size to catchment area, annual HL, TSS load and concentration were determined by Pearson correlation. A general linear model, with seasonal hydraulic and particle load as variables for each wetland, was used to explain sediment deposition in the different seasons. Individual wetlands in which the same water sampling technique was used at the inlet were compared pairwise between years. The wetlands were also divided into deep areas (>1 m) and shallow areas (<0.5 m) to test for differences in sediment deposition, accumulation and resuspension between wetland sections, using a mixed model with depth as fixed factor and year as replicate. Since repeated measurements of accumulation on each plate took place for the same wetland, correlations between the plates were modelled using a spatial power correlation structure, where the spatial location was described by transect and side. A similar mixed model was used to evaluate differences between transects within a wetland and to test for differences between the plates within transects. Any differences between the sum of sediment accumulated on plates during two years and the sediment depth measured with a stick in year 2 in the Nyb wetland were tested with a two-sample *T* test. Because of non-normality, the data were log-transformed prior to analysis, and analyses were performed using Minitab 16 Statistical Software.

## Results

### Hydraulic characteristics and particle concentrations

The annual modelled HL in Wig was significantly higher than that measured in the other wetlands (Table 2). Events with high TSS concentrations occurred 5–8 times per year (all year round). Estimated high loads of TSS determined using the flow-proportional technique were confirmed with independent measurements using online sensors in Ber wetland in later years (Supplementary Material). The mean inflow TSS concentration in the three water quality monitoring wetlands decreased slightly in the order: Ski > Nyb > Ber. However, as HL was higher in Nyb, the highest TSS load was observed for that wetland (24 kg m^−2^ year^−1^).

**Table 2:** Number (No.) of observed years water flow (runoff and discharge) into the wetlands, hydraulic load (HL), mean inflow concentration and load of total suspended solids (TSS) from water sampling in the Nyb, Ber, Ski and Wig wetlands related to the wetland area. Mean annual sediment deposition (Depos), accumulation (Accum), area-specific and relative resuspension (Resusp) from sediment sampling. ^a, b, c, d^ = significant difference (*p* < 0.05) between wetlands with flow-proportional water sampling at the inlet and studied for more than 2 years. Last two columns give estimated P accumulation on plates and retention (P ret) from water samples. For further information and discussion, see text

	*N* ^†^	Water flow	HL	TSS_in_	TSS load	Depos	Accum	Resusp	Resusp	Paccum^X^	P ret
		(mm year^−1^)	(m year^−1^)	(mg L^−1^)	(kg m^−2^ year^−1^)				(%)	(kg ha^−2^ year^−1^)	(%)
Nyb	2	274	120	137	24	190	23	170	87	240^β^	−4
Ber	4^‡^	218	70^b^	108	10	30^a^	6^a^	24^a^	83^a^	90	36
Ski	3	208	60^b^	143	15	10^b^	2^b^	8^b^	84^a^	25	n.d.
Wig	3^+^	176^+^	400^+a^	n.d.	n.d.	4^c^	1^b^	3^c^	77^a^	10	n.d.

^X^ From Johannesson et al. (2015)

^β^ Adapted from Kynkäänniemi (2014)

^†^ Number of years is also the number of samples in the statistical analyses

^‡^ Water sampling only for 3.5 years

^+^ No measurements of water flow in this study, modelled runoff and hydraulic load based on runoff data from SMHI’s Water Web

### Sediment deposition, accumulation and resuspension

#### Annual sediment deposition, accumulation and resuspension

Annual sediment deposition differed between all wetlands and decreased in the order: Nyb > Ber > Ski > Wig (Table 2). In the newly constructed Nyb and Ber wetlands, annual sediment deposition was eight-fold and three-fold higher, respectively, than the measured TSS load, but slightly lower than the load in the Ski wetland. Sediment accumulation followed the same order as deposition for all wetlands (Table 2), but in all wetlands annual accumulation amounted to only 13–23% of the amount deposited in the traps. The estimated mean annual area-specific resuspension was considerable and differed significantly between all wetlands.

Annual deposition and accumulation were significantly positively correlated with water flow (*p* < 0.05) including modelled water flow for Wig, but not significantly and with only a weak tendency (0.05 < *p* < 0.1) to the LW coefficient (Table 3a). With only measured flow (excluding the Wig wetland), there was a positive correlation with HL for both deposition and accumulation (Table 3b).

**Table 3:** Pearson correlation coefficients for relationships between sediment deposition (Log Depos), accumulation (Log Accum) and resuspension (Log Resusp; Rel. Resusp) and wetland relative size to catchment area (A_w_:A_c_) and wetland length-to-width ratio (L:W), as well as hydraulic load (HL), runoff (Q), load and inflow concentration of total suspended solids (TSSload and TSSin). (a) All wetlands and with modelled Q for the Wig wetland (*n* = 12). (b) With only measured data and Wig excluded (*n* = 9)

	(a) All wetlands (modelled Q for Wig)						(b) Only measured data		
	Log Accum	Rel. Resusp	A_w_:A_c_	L:W	Q	HL	HL	TSS_load_	TSS_in_
Log Depos	0.97*	0.30	0.41	0.53	0.70*	−0.21	0.94*	0.53	0.26
Log Accum		0.02	0.33	0.56	0.62*	−0.37	0.76*	0.50	0.30
Log Resusp			0.43	0.52	0.71*	−0.45	0.77*	0.54	0.25
Rel. Resusp			0.45	0.01	0.41	−0.44	0.27	0.19	−0.20

* Significant difference (*p* < 0.05) between factors

**Fig. 3:**
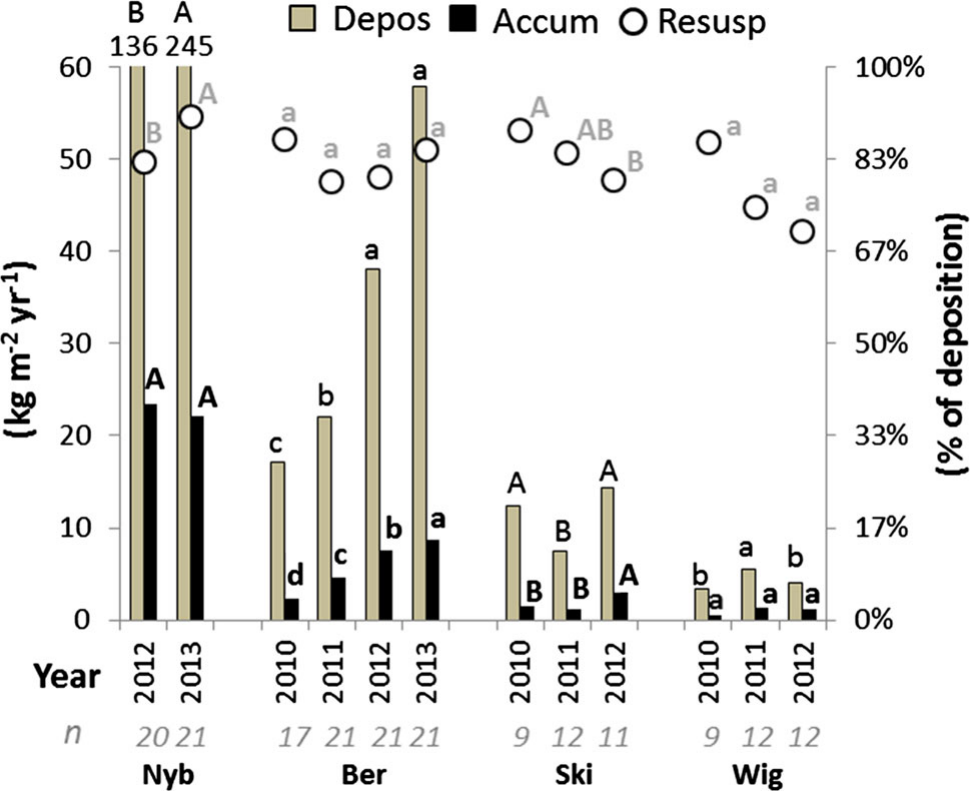
Mean sediment deposition (Depos) and accumulation (Accum), and relative resuspension (% of deposition) in the Nyb, Ber, Ski and Wig wetlands. For each wetland, annual means with different letters (*A* and *B* or *a*, *b*, *c* and *d*) are significantly different (*p* < 0.05). *n* = number of samples

Sediment accumulation increased significantly between the years in Ber (Fig. 3). The sediment deposition was also higher in the third and fourth year than in the first two years, but there was no difference between the years in estimated relative resuspension. In contrast, no difference in sediment accumulation between the years was observed in Nyb and Wig, even though they were also newly constructed. In Nyb, the deposition was almost twice as high in the second year and hence the estimated relative resuspension was also higher in that year. In the older Ski wetland, the sediment accumulation was higher in the last year than in the two previous years, and the calculated relative resuspension was lower.

#### Seasonal sediment deposition

**Fig. 4:**
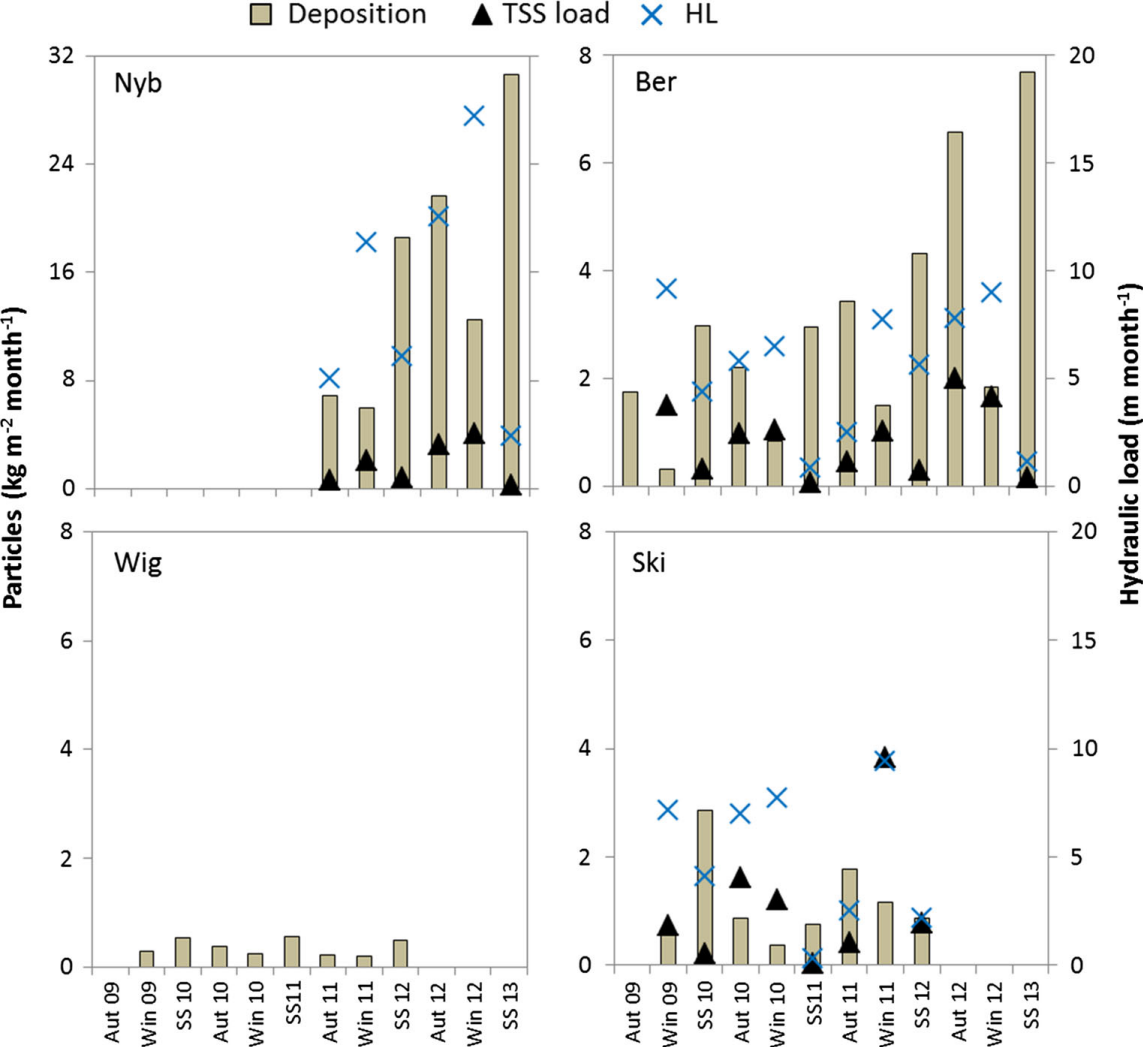
Seasonal sediment deposition estimated by sediment traps (bars) in the Ber, Ski, Wig and Nyb wetlands. Measured hydraulic load, shown as crosses, and load of total suspended solids (triangles) were estimated from water samples for all wetlands except Wig, where no water samples were taken. Note the different scales on the particle axis for Nyb

The TSS load entering the wetlands was always higher during winter (including snowmelt) than in spring–summer (Fig. 4). However, the seasonal sediment deposition in traps was not correlated with seasonal variables HL, maximum water flow, TSS load and TSS concentration. There was significantly higher sediment deposition in spring–summer than in winter in the Ber wetland, while in Wig sediment deposition was higher in spring–summer than in autumn. In general, the measured deposition of particles was higher than the estimated load of TSS during spring–summer in the three wetlands with water quality data, except in spring–summer 2012 in Ski. The arable catchment in Ski was under ley but it was ploughed down at the end of the summer 2011, which might explain the extremely high load of particles during the following winter. In the Ber and Nyb wetlands, the deposition was equal to, or exceeded, the TSS load during most seasons. In Nyb, constructed in a ditch, the seasonal deposition was positively correlated with the seasonal FFI (*r* = 0.69), whereas no such correlation was found for the other two wetlands (Ber and Ski) with tile-drain inlets.

### Sediment deposition, accumulation and resuspension in different wetland areas

**Fig. 5:**
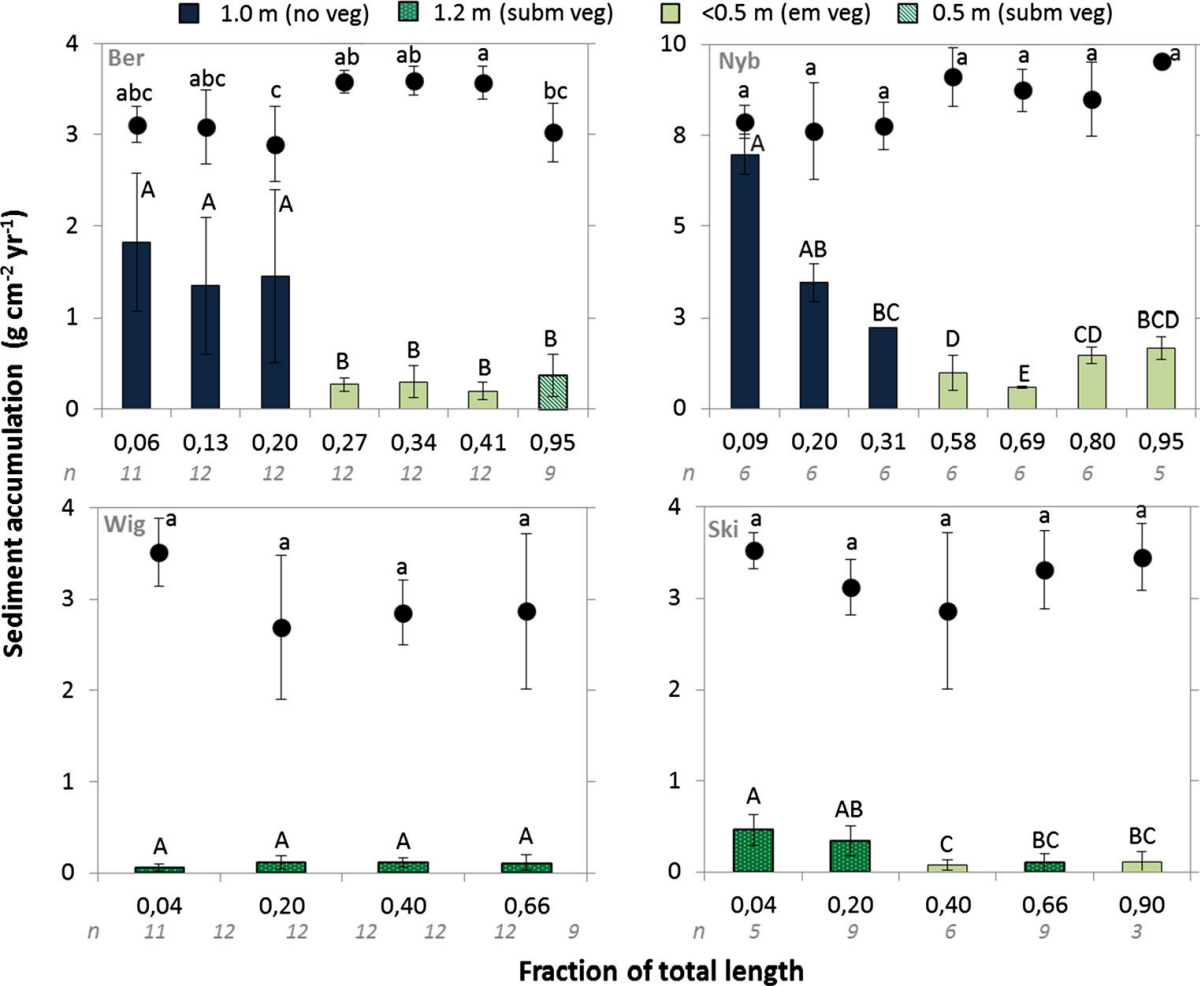
Mean sediment accumulation for each sampling transect (bars) and relative resuspension (circles) with standard deviation as error bars. The wetlands are divided into deep (>1 m) and shallow (<0.5 m) areas. Deep areas are blue (no vegetation) and dark green (submerged vegetation). Shallow areas are light green (emergent vegetation) and green stripes (submerged vegetation). Means within each wetland with different letters (*A*, *B*, *C* and *D* or *a*, *b* and *c*) indicate significant differences (*p* < 0.05) between transects. *n* = number of samples

**Fig. 6:**
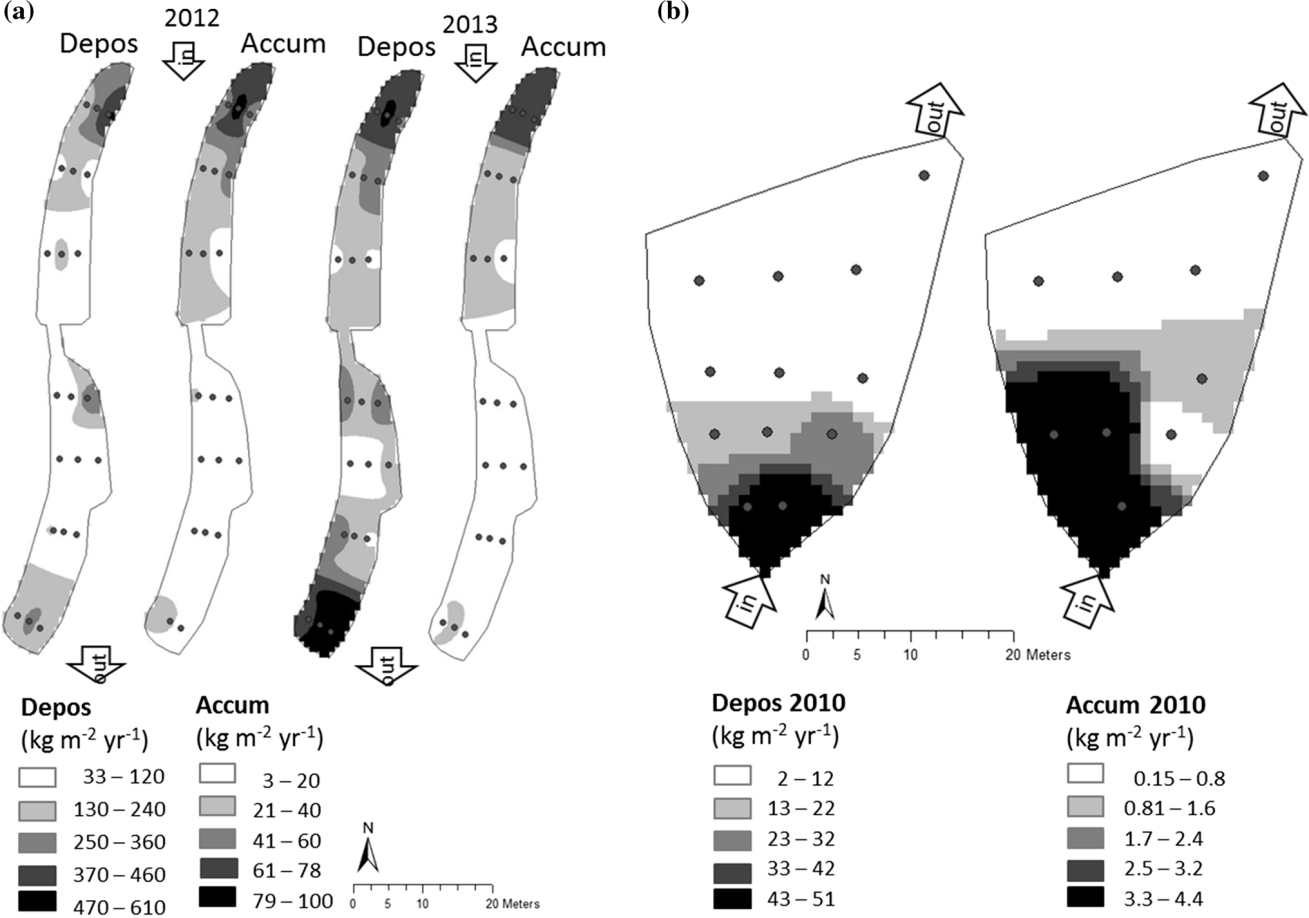
Sediment deposition (traps) and accumulation (plates) (**a**) in the Nyb wetland in 2012 and 2013 and (**b**) in the Ski wetland in 2010

Overall, sediment accumulation and estimated resuspension were higher in the initial wetland area than in the remaining part of the wetlands (Fig. 5). Most of the total sediment accumulation was found in the initial 20% of the total wetland area in Nyb (79 ± 2%), Ber (84 ± 4%) and Ski (83 ± 4%), but only (43 ± 18%) in the Wig wetland. On 26 December 2011, a 150 m road ditch draining to the stream just upstream of the Nyb wetland was dredged by excavating the banks and bottom. A large amount of particles was transported and could be seen to have settled at the entrance to the deep basin, before the first transect. Subsequent movement of this sediment within the wetland was indicated by much higher deposition in the final wetland section in 2012 than in 2011 (Fig. 6a). However, a corresponding loss of sediment from the inlet section could not be detected, as there was no significant difference between the sum of sediment accumulation on the plates in the deep section during these two years and the total depth measured by a stick in any of the first three transects. Mean sediment accumulation in that part was 15 cm.

There was no significant difference in sediment deposition in traps, accumulation on plates or resuspension between the right, middle and left sampling points within the different transects in any of the wetlands. However, spatial interpolation of sediment deposition and accumulation in Ski in 2010 indicated higher sediment deposition, but lower sediment accumulation along the right side of the wetland. This would indicate higher resuspension along this side, i.e. the shortest flow path from inlet to outlet (Fig. 6b).

### Phosphorus concentrations in accumulated sediment and related to suspended solids

Phosphorus at wetland inlet and outlet was mainly in particulate (PP) form, especially in Nyb and Ski. Phosphorus concentrations, especially PP, also demonstrated a linear relationship with TSS concentrations for all wetlands, with a high coefficient of determination (Table 4). The TP/TSS ratio was relatively large for Ber wetland, in an area where hotspots with very high soil P concentrations have been documented (Parvage et al. 2011). The ratio was also large for the small Wig wetland with its moderate soil P concentration. In contrast to TP/TSS ratio, mean PP/TSS ratio was rather similar for the four wetlands (Table 4).

**Table 4:** Mean number (*N*) of samples and estimated linear regression between concentrations of total phosphorus (TP) and total suspended solids (TSS) and between particulate phosphorus (PP) and TSS in inlet and outlet (all in mg L^−1^) with the linear regression slope and coefficient of determination (*r* ^2^). Estimated P concentrations in TSS and below actual measured (geographical integrated mean) TP concentration in accumulated sediment in the four wetlands are also shown

	*N*	Linear regression TP and TSS		TP/TSS	Linear regression PP and TSS		PP/TSS
		Slope	*r* ^2^	(%)	Slope	*r* ^2^	(%)
Water							
Nyb_inlet_	44	0.0011	0.83	0.17	0.0028	0.85	0.12
Nyb_outlet_	44	0.0012	0.87	0.18	0.0011	0.85	0.11
Ber_inlet_	88	0.0015	0.71	0.28	0.0014	0.81	0.15
Ber_outlet_	88	0.0014	0.81	0.25	0.0013	0.88	0.15
Ski_inlet_	54	0.0011	0.97	0.16	0.0010	0.97	0.11
Ski_outlet_	54	0.0010	0.82	0.18	0.0009	0.85	0.13
Wig_inlet_	23	0.0010	0.77	0.26	0.0008	0.89	0.13
Wig_outlet_	23	0.0011	0.72	0.26	0.0009	0.86	0.14
Sediment plates							
Nyb	42	–	–	0.10	–	–	–
Berg	42	–	–	0.15	–	–	–
Ski	24	–	–	0.13	–	–	–
Wig	24	–	–	0.09	–	–	–

In Nyb, with a high HL, the high sediment accumulation and high resuspension was found to be accompanied by low net retention estimated from sampled water (Table 2). In contrast, moderate HL and TSS load were accompanied by moderate accumulation and 36% P retention in the Ber wetland.

## Discussion

The highest annual particle accumulation observed in the present study, 23 kg m^−2^ year^−1^ in Nyb, was higher than measured accumulation (1–11 kg m^−2^ year^−1^) in four other Swedish wetlands investigated by Johannesson et al. (2015). It was more similar to the particle retention estimated from inflow–outflow balance (15–75 kg m^−2^ year^−1^) in four constructed wetlands receiving high hydraulic loads (440–1200 m year^−1^) in Norway (Braskerud 2001b). However, the results presented here suggest that the accumulation did not solely represent retention of inflowing particles, particularly in the new wetlands. The measured accumulation on plates in Nyb and Ber (Table 1) would represent extremely efficient retention of measured TSS load (96 and 60%, respectively), which is unlikely. The most striking difference was for Nyb, where the water quality measurements for inlet compared with outlet indicated that the wetland was a source of particles (Kynkäänniemi 2014) for the downstream water, rather than a sink. However, retention calculated based on data from the shallow section was estimated at 4.3 kg m^−2^ year^−1^, suggesting substantial erosion and resuspension of material from the first deeper section to the second shallow section.

Even though annual accumulation was higher in wetlands with high deposition, it only represented 13–23% of the amount deposited in traps, suggesting that internally generated particles also settled temporarily in the wetlands. This is supported by the observation that the deposition was equal to or much higher than the measured annual TSS load in the three water-monitored wetlands (Table 1). The edge of the traps extended approximately 2 cm above the sediment surface to avoid capturing particles that moved close to the sediment surface due to bottom erosion, but despite this, the sediment deposition was higher than the TSS load in most seasons in both Ber and Nyb (Fig. 3). This suggests that the wetland bottom was quite prone to erosion during the first years after construction. However, the deposition of such particles could have been overestimated because the turbulence and flow velocity in the trap cylinders are low, allowing particles to resettle faster than they would do outside the cylinders (Kozerski and Leuschner 1999), This would explain some of the large discrepancy between measured deposition and accumulation on plates. In addition, the measured accumulated material on plates may have comprised some internally generated organic material from higher plants and algae, particularly in the shallow parts with more plants (Johannesson et al. 2015). However, in this study this represented only a minor proportion, as the accumulation in the shallow sections was less than 20% of the total.

The much smaller difference between deposition and TSS load in the older Ski wetland (Fig. 3) provides indirect support for the suggestion that erosion contributed a large amount of the particles caught in the traps in the newly constructed Ber and Nyb wetlands. Fennessy et al. (1994) also reported much higher sediment deposition than inflow of suspended particles in four wetlands receiving pumped river water, and attributed this to resuspension and internal production of organic matter. Resuspension was not measured in that study, but the authors speculated that the declining water depth (especially in a low-load wetland) as the growing season progressed may have led to increased sediment recirculation from wind-generated waves. Dieter (1990) studied resuspension differences between open water and areas with emergent vegetation in two large (total area > 4000 ha) wetlands (mean depth 0.6 and 1.0 m) and found approximately three-fold higher resuspension in the open water during three summer months. They attributed this difference to the physical influence of wind-generated waves. In one wetland with fine (silt) sediments, the resuspension was equal to 30 kg m^−2^ year^−1^ in the emergent vegetation and 78 kg m^−2^ year^−1^ in open water. In the present study, the resuspension was lower in Ber, Ski and Wig (3–24 kg m^−2^ year^−1^), but much higher in the Nyb wetland (170 kg m^−2^ year^−1^), suggesting considerable physical disturbance of the bottom sediment in the latter wetland. Nyb was also the only wetland where the seasonal deposition was well correlated with FFI, indicating that more particles were eroded and transported during periods of rapid flow variations. Large flow variations mean more events with high water velocities, which should generally cause more erosion, in a corresponding way as in small streams (Veihe et al. 2010). Much erosion was visible, especially from the large heap of particles at the Nyb inlet after dredging the ditch upstream. Movement of those particles was indicated by higher deposition at the outlet in the second year (Fig. 6a), and the fact that TSS concentrations at the water sampling point between the deep and shallow area exceeded the inlet concentrations (Kynkäänniemi, 2014).

The importance of erosion is also indicated by the observation that the deposition increased steadily over the seasons in the new Ber and Nyb wetlands, except during each winter period, when the hydraulic loads were high (Fig. 4). As the amount of accumulated material in the wetlands gradually increased, more material could be eroded and deposited during periods of high water velocities.

Problems with shore and bottom erosion could be expected to be aggravated if a wetland is constructed in a ditch with running water, like the Nyb wetland. For example, Kronvang et al. (1997) and Laubel et al. (2003) demonstrated that in a Danish agricultural stream, bank/bed erosion accounts for 40–80% of TSS, with the largest contribution coming from the lower part of the bank or stream bottom. Kronvang et al. (1997) estimated that approximately 20 kg TSS erodes from each metre length of stream per year. In Nyb, the problems may have been worsened by excavation started under frozen conditions. In contrast, Ber was constructed during a dry summer and by digging beside a drainage culvert, probably resulting in more consolidated bottom, which could be the reason for the apparently lower erosion in Ber than in Nyb (lower difference between deposition and TSS load). Furthermore, a low water level in summer, which was especially apparent in Nyb, might have been followed by accelerated erosion when water level increased again in autumn.

More than 50% of retained particles settled in the initial 30% of the wetland area (inlet delta and deep area) in the study by Braskerud et al. (2000), and the annual accumulation decreased with distance from the inlet. In the present study, even more (approximately 80%) of the total particle accumulation was found in the initial 20% of the wetland area in three of the present wetlands. The percentages of deposited and accumulated particles that settled in the inlet area of Ski were similar, while in Nyb and Ber lower proportions were deposited in the initial area (Table 3), again indicating re-deposition of eroded particles further down in the newly constructed wetlands. Brueske and Barrett (1994) also found that estimated particle accumulation near the inflow was extremely high, 33 and 16 cm year^−1^ in a high-loaded and low-loaded wetland, respectively. These values are similar to those found for the first transect in Nyb.

In the fourth wetland, Wig, only 40% of the total accumulation was found close to the inlet, and relative deposition was higher than accumulation in this part (50% of the total amount). This indicates resuspension of accumulated particles at the inlet, but no re-deposition further down in the wetland. This provides support for the hypothesis suggested by Johannesson et al. (2015) that the HL to the Wig wetland (400 m year^−1^) is too high for efficient particle accumulation. The present study showed that the Wig wetland was not only too small to act as a trap for inflowing particles, but also for most particles that may have been resuspended and/or eroded from the sides and bottom, as the deposition was also very low (only 4 kg m^−2^ year^−1^).

Braskerud (2001a) suggested that wetlands should be shallow for a short vertical settling distance. However, with a fixed wetland width, reducing the distance between sediment and water surface also increases the water velocity and could thereby increase the risk of resuspension. In Ber, the relative resuspension was indeed lower in the deep area than in the shallow area. This could also have been caused by settling of larger particles, less prone to resuspension, close to the inlet, while smaller particles probably settled in the shallow vegetated areas. A study of the particle size distribution and their P concentrations in the different transects would be necessary to confirm this.

Phosphorus may be enriched by selective erosion of P from the soil (Sharpley 1980). The study area has clay soils which are tile-drained. A previous study revealed that small clay colloids dominate and larger particles are rare in the water from drain pipes and in stream water in clay soil areas (Ulén 2004). In the present study, there was only a small variation between the four wetlands in terms of the yearly mean ratio between PP and TSS (0.11–0.15%) at the inlet (Table 4). The differences between the P concentrations at the wetland inlet and outlet were also minor. There were no indications of a spatial gradient whereby larger particles settled more easily close to the inlet and changed the P content in the accumulated sediment. In addition, these estimated P concentrations in TSS in water was just slightly higher than the spatial mean P concentration in accumulated sediment (0.09–0.15%). Hence based on the present study, any disproportionality in settling of larger particles seems to be of limited importance for the amount of P ultimately retained in the wetlands studied.

The positive correlation of sediment deposition and accumulation with measured HL for the three wetlands with modest HL agrees well with other studies (Kadlec 1995). Within the HL interval 60–120 m year^−1^, the corresponding increase in particle load resulted in higher area-specific retention in the wetlands studied here. Despite lower HL, the particle load was higher in Ski than in Ber, which should have resulted in higher sediment retention according to Braskerud (2003). However, both sediment deposition and accumulation were significantly higher in Ber (Table 1).

There was no positive correlation between sediment accumulation and wetland shape (i.e. L:W) in the present study. With no significant difference in sedimentation or resuspension between the sampling points within transects, the wetlands may be assumed to be hydraulically efficient, having an even distribution of the sediment load within transects. However, in Ski the results from areal interpolation of plate and trap data revealed higher sediment deposition, but lower accumulation along the right side from inlet to outlet in the Ski wetland. This indicated preferential flow, which could have resulted in higher resuspension and erosion, thus lowering accumulation during high flows. In addition, during low flow periods, the inflowing particles would settle preferentially along the right side. The preferential sediment deposition pattern was probably due to the shape (low L:W), in combination with the location of the inlet and outlet. Furthermore, compared with Ber, where water was always flowing, the flow variations in Ski were greater, with zero inflow during summer. This lowered the water level to the extent that the shallow middle section was dry. A possible consequence of this could be erosion of settled particles not protected by a standing water layer during the first autumn rain event.

## Conclusions

Internal erosion and sediment movement may be of seasonal importance in newly constructed wetlands in clay soil areas, as sediment deposition mostly exceeded the load of suspended particles in spring–summer season in three out of four wetlands in this study.

More than 80% of the total sediment accumulation occurred in the initial areas (representing the first 20% of the total wetland area), except in the smallest wetland, which demonstrates the importance of a deep area at the inlet for trapping particles and associated P.

The difference in sediment deposition and accumulation between wetlands could not be explained by relative resuspension, as this did not differ between wetlands. Sediment accumulation was strongly correlated with deposition, but amounted to only 13–23% of the annual deposition in traps. This further indicates that internal erosion and resuspension occurred, particularly in the recently constructed wetlands.

Sediment deposition and accumulation were positively correlated with HL in three of the wetlands, but not in the wetland with the highest HL (400 m year^−1^), where both sediment deposition and accumulation were low. This indicates that there is an upper limit for HL if a wetland is to function as sediment trap.

Seasonal variations in deposition in one wetland with extremely high deposition were correlated with a fast-flow index, suggesting that flow variations may be particularly important when wetlands are located in ditches.

High accumulation and resuspension was demonstrated to accompany by negative P retention in one wetland, while moderate accumulation and HL were accompanied by 36% P retention in another wetland.

## Electronic supplementary material

Below is the link to the electronic supplementary material.

Supplementary material 1 (PDF 103 kb)
