# Supplementary material for: Particle deposition, resuspension and phosphorus accumulation in small constructed wetlands
Source: Ambio. 2017 Nov 21;47(Suppl 1):134–45. doi: 10.1007/s13280-017-0992-9 (PMC5722750; doi:10.1007/s13280-017-0992-9)
Supplement: Supplementary file 1 — Supplementary material 1 (PDF 103 kb) [file 13280_2017_992_MOESM1_ESM.pdf]

Ambio

Electronic Supplementary Material
This supplementary material has not been peer reviewed.

Title: **Particle deposition, resuspension and accumulation in different designed wetlands**

Authors: Pia Geranmayeh, Karin M Johannesson, Barbro Ulén, Karin S. Tonderski

Vegetation in the wetlands

The shallow areas in Ber and Nyb were planted mainly with greater pond sedge (*Carex riparia* L.) and yellow iris (*Iris pseudacorus* L.) in the years after construction (2009 and 2011). The vegetation coverage in Ber was sparse in the first few years but dense (>50%) by summer 2012 and even denser by summer 2013. In Nyb, the vegetation established more quickly than in Ber and was dense already in the second year. Ski was constructed in 2002 and dense blankets of broad-leaved pondweed (*Potamogeton natans* L.) soon covered the deep sections. The shallow area between them, as well as around the shores, was dominated by cattail (*Typha latifolia* L. and *Typha angustifolia* L.). The Wig wetland was constructed in spring 2009 as a single pond and patches of cattail (*Typha latifolia* L.) and sedge (*Carex* sp.) was established along with water plantain (*Alisma plantago-aquatica* L.), common duckweed (*Lemna minor* L.) and whorled water milfoil (*Myriophyllum verticillatum* L.).

Loads of TSS and P to wetlands at high-flow events

a) b)

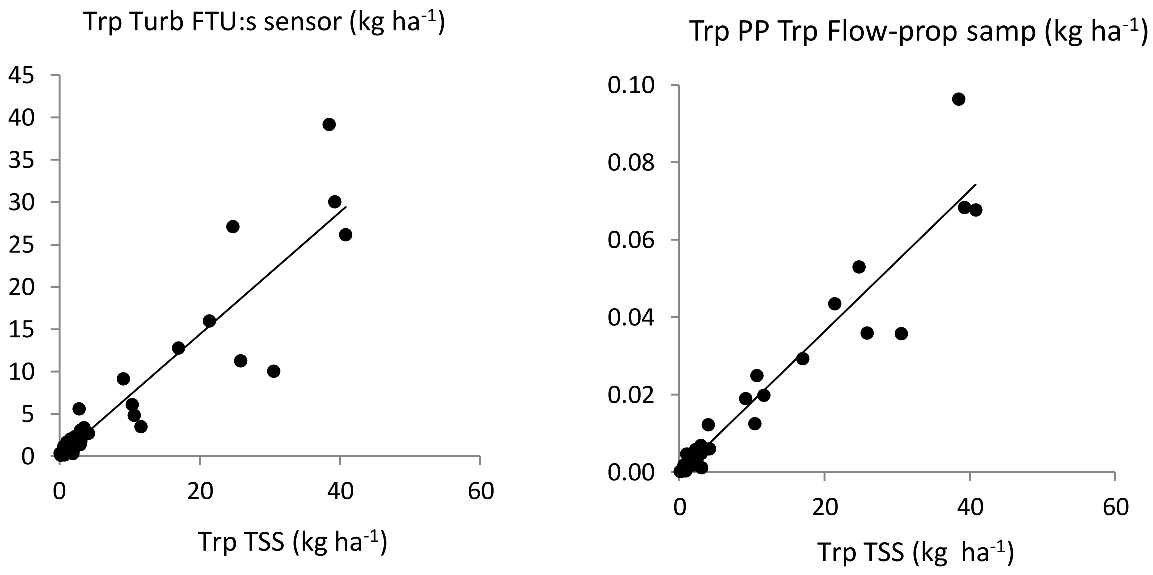

Figure S1a) Regression line between biweekly loads of a) transport based on turbidity values (FTU:units) from a sensor with recorded data stored hourly and total suspended solids from composite flow-proportionally sampling and b) particulate phosphorus and total suspended solids at the inlet of Ber wetland. In 2013/2015 at eight periods with high loads (> 15 TSS kg ha^{-1}), turbidity values indicated both more than 45% and less than -31% particle transport than expected from the regression line. Similarly, both high (0.25%) and low (0.12%) PP concentrations related to TSS values were estimated in periods including high-flow events.
